# Determinants and Causes of Neonatal Mortality in Jimma Zone, Southwest Ethiopia: A Multilevel Analysis of Prospective Follow Up Study

**DOI:** 10.1371/journal.pone.0107184

**Published:** 2014-09-18

**Authors:** Gurmesa Tura Debelew, Mesganaw Fantahun Afework, Alemayehu Worku Yalew

**Affiliations:** 1 Department of Reproductive Health and Health Service Management, School of Public Health, Addis Ababa University, Addis Ababa, Ethiopia; 2 Department of Epidemiology and Biostatistics, School of Public Health, Addis Ababa University, Addis Ababa, Ethiopia; Vanderbilt University, United States of America

## Abstract

**Background:**

Ethiopia is among the countries with the highest neonatal mortality with the rate of 37 deaths per 1000 live births. In spite of many efforts by the government and other partners, non-significant decline has been achieved in the last 15 years. Thus, identifying the determinants and causes are very crucial for policy and program improvement. However, studies are scarce in the country in general and in Jimma zone in particular.

**Objective:**

To identify the determinants and causes of neonatal mortality in Jimma Zone, Southwest Ethiopia.

**Methods:**

A prospective follow-up study was conducted among 3463 neonates from September 2012 to December 2013. The data were collected by interviewer-administered structured questionnaire and analyzed by SPSS V.20.0 and STATA 13. Verbal autopsies were conducted to identify causes of neonatal death. Mixed-effects multilevel logistic regression model was used to identify determinants of neonatal mortality.

**Results:**

The status of neonatal mortality rate was 35.5 (95%CI: 28.3, 42.6) per 1000 live births. Though significant variation existed between clusters in relation to neonatal mortality, cluster-level variables were found to have non-significant effect on neonatal mortality. Individual-level variables such as birth order, frequency of antenatal care use, delivery place, gestation age at birth, premature rupture of membrane, complication during labor, twin births, size of neonate at birth and neonatal care practice were identified as determinants of neonatal mortality. Birth asphyxia (47.5%), neonatal infections (34.3%) and prematurity (11.1%) were the three leading causes of neonatal mortality accounting for 93%.

**Conclusions:**

This study revealed high status of neonatal mortality in the study area. Higher-level variables had less importance in determining neonatal mortality. Individual level variables related to care during pregnancy, intra-partum complications and care, neonatal conditions and the immediate neonatal care practices were identified as determinant factors. Improving antenatal care, intra-partum care and immediate neonatal care are recommended.

## Introduction

Globally, about 6.6 million children die before their 5^th^ birthday each year. About 5 million of this occurs in the first year of life and nearly 3 million die within the first 28 days of birth. This indicates that about 44% of under-five deaths and 60% of infant deaths are accounted by the neonatal mortality. Moreover, the share of neonatal mortality from the under-five death rose from 37% in 1990 to 44% in 2012 [1]. This clearly points out that it is difficult to achieve the desired millennium development goal (MDG_4_) target for the two-thirds reduction of child mortality by 2015 without particular focus on neonatal mortality.

More than 98% of these deaths occur in developing countries. Sub-Saharan Africa has the highest risk of death in the first month of life and among the regions showing the least progress in reducing the neonatal mortality rate. Most of these deaths are caused by infectious diseases, pregnancy-related complications, delivery-related complications, including intra-partum asphyxia, birth trauma and premature birth which can easily be prevented [1], [2].

In Ethiopia, neonatal mortality rate (NMR) has long been very high. In spite of many efforts by the government and other stakeholders, non-significant and very sluggish decline has been achieved in the last 15 years. The neonatal mortality rate for the years 1991–1995, 1996–2000, 2001–2005 and 2006–2011 were 46, 42, 39 and 37 per 1000 live births, respectively [3], [4], [5]. Moreover, about 63% of infant deaths in the country occur during the first month of life. Thus, accelerated reduction in neonatal mortality is increasingly critical for progress towards the MDG_4_.

To do this, identifying the determinants and causes of neonatal mortality at the local context is very crucial and timely issue. However, many of the neonatal deaths happen at home and unrecorded that make obtaining sampling-frame and sources of data very difficult. As a result, studies on this topic including verbal autopsies (VAs) are limited in Ethiopia. Moreover, the very few available studies are only facility-based and cross-sectional in design, which are not the preferred designs to establish causal relationships necessitating community-based prospective study supported by verbal autopsy (VA).

Therefore, this longitudinal community based study aimed to fill these gaps by determining the status of neonatal mortality and identifying the determinants and causes at different levels by applying multilevel analysis. The findings of the study will also be used as inputs for policy makers and program implementers at national as well as regional levels to design evidence-based intervention strategies to tackle the problems of neonatal mortality.

## Methods and Materials

### Study design and setting

This study was a community-based prospective follow up conducted in Jimma Zone from September 2012-December 2013. Jimma Zone is one of the 17 Zones of the Oromia Regional State of Ethiopia having a total of 17 rural districts and two town administrations. According to the 2007 national population and housing census, the Zone has a total population of 2.6 million, of which 88.7% are rural residents [6], [7].

### Sample size and sampling technique

The minimum required sample size for this study was determined by using Epi-Info V.3.5.1 by considering two sample comparisons of proportions based on the following assumptions. The outcome variable was neonatal mortality. Among all the determinants of neonatal mortality considered, educational status of mothers was found to give the largest sample size. Based on this, the prevalence of neonatal mortality among mothers having educational status of secondary or above was estimated to be 4.0% (P_1_ = 0.040) and among those who didn't attend secondary education was to be 8.1% (P_2_ = 0.081) [8]; 95% level of confidence and 80% power were considered. A ratio of 1∶3 was used (r = 3). As multistage-clustered sampling method was used, a design effect of 2 was considered. Finally, 10% was added for non-responses and miss-to-follow up and the final sample size became 3604.

Multistage-clustered sampling technique was used to identify a cohort of pregnant women to be enrolled in the follow up for the study. At first stage, the Zone was stratified as rural districts (17 in number) and town administrations (2 in number, Jimma and Agaro). Then, by considering time and logistics, 5 districts (30%) were selected by simple random sampling from the 17 districts. At second stage, all the selected 5 districts were clustered by ‘Kebeles’ (A ‘kebele’ is the smallest administrative unit having 5000 population in average) and stratified in to urban and rural ‘Kebeles’.

Then, by simple random sampling method, 9 rural ‘Kebeles’ and 2 urban ‘Kebeles’ were selected from each selected district. This number of clusters (‘kebeles’) was determined based on expected number of pregnant women per ‘Kebele’. Jimma town administration and Agaro town administration have 13 and 5 ‘Kebeles’, respectively and all were included purposefully. With this, a total of 73 Clusters (‘Kebeles’) were included in the study. Then, for all selected ‘kebeles’, pregnant women were enumerated by using house-to-house visit and all obtained were enrolled in the study (Figure S1).

### Measurements

The dependent variable for this study was neonatal mortality and the independent variables were divided into two levels. Level 2 (higher-level variables) included community or cluster level variables such as place of residence, access to health centers and access to hospitals. Level 1 (lower-level variables) included individual and household characteristics such as: socio-demography, wealth quintiles, maternal obstetric factors, maternal health care use, conditions of labor, characteristics of the neonates and neonatal care practices. The detail descriptions and measurements are given below (Table 1).

**Table 1:** Description of variables and measurement for the study, Jimma Zone, Southwest Ethiopia, September 2012-December 2013.

Variables	Descriptions	Measurements
Dependent variable		
Neonatal mortality	Death of the infant before 28 completed days	Neonates died before 28 days were categorized as neonatal death and coded as ‘1’, those survived 28 days were coded as ‘0’
Level-2 predictor variables	Communal (kebele) characteristics	
Place of residence	The usual place of residence where the woman lives	Urban kebele was coded as ‘1’ and rural kebele was coded as ‘0’.
Average distance from health centre	Approximate distance of respondent's home from the nearest health centre on foot in munities as reported by respondent.	Average distance was computed for each kebele and dichotomized as ‘≤2 hours’ and ‘>2 hours’
Average distance from Hospital	Approximate distance of respondent's home from the nearest hospital on foot in munities as reported by the respondent.	Average distance was computed for each kebele and categorized as ‘≤2 hours’, ‘>2–12 hours’ or ‘>12 hours’
Level-1 predictor variables	Individual and household characteristics	
Age	Age of women at interview in completed years	Categorized in to 7 groups by five-years interval, which later recoded in to three categories: ‘<20’, ‘20–29’ or ‘>29’
Ethnicity	The ethnic background of the respondent	Each ethnicity was entered and later recoded as ‘Oromo’ and ‘Others’. Others were merged because they were very few for logistic regressions.
Religion	The religious background of the respondent	Each religion was entered and later recoded as ‘Muslim’ or ‘Others’. Others were merged because they were very few for logistic regressions.
Educational status	Highest level of education attained by the respondent and her husband	Categorized in to 4 groups as ‘no Formal Education’, ‘primary (1–8)’, ‘Secondary (9–12)’ and ‘tertiary (12^+^)’.
Occupational status	Current employment status and specific occupation of respondent and her husband	Categorized as ‘housewife’ (‘farmer’ for husbands), ‘employed’, ‘merchant’ and ‘others’.
Wealth quintiles	Using EDHS questionnaire, house hold assets ownership were assessed and wealth index was computed by using principal component analysis	The wealth status was categorized in to five groups and ranked from poorest to wealthiest quintile.
Birth order	Number of births a woman ever had including current birth	The responses was categorized in to three categories as: ‘1^st^ birth order’, ‘2^nd^–4^th^’ and “≥5^th^ birth order’
Preceding birth interval	The duration between the current birth and the preceding birth in months.	The responses were categorized in to three as: ‘<24 months, ‘24–48 months’ and ‘‘>48 months. First birth orders were categorized as ‘Nuliparous.’
Birth preparedness and complication readiness	A package of interventions composed of composite measure of 5 variables (planed to save money, planed to arrange transport, identified place of delivery, identified skilled attendant and identified blood donor)	Composite variable was computed by adding the five responses. Women who scored 3 or more ‘Yes’ responses were categorized as ‘prepared’ otherwise ‘not prepared’
ANC frequency	Having health facility visit for pregnancy check up by skilled attendants during pregnancy.	Categorized in to three: ‘No ANC visit at all’, ‘1–3 ANC visits’ and ‘≥4 ANC visits’
Place of delivery	The place where the neonate was born	Categorized as ‘home’, ‘Hospital’ and ‘Health centre’.
Attendant of delivery	The person who assisted the mother during delivery	Those who have trained to the level of Diploma and above was categorized as “skilled attendants”, those who didn't train at all, TBAs/TTBAs and HEWs were categorized as ‘Unskilled attendants’
Gestation age at birth	Approximate GA at birth by woman's own report in weeks	GA of <37 weeks were categorized as ‘Premature birth’ and GA of ≥37 weeks were categorized as ‘Mature birth’.
Premature rupture of membrane (PROM)	Leakage of fluid before the onset of labor and the duration it stayed before the onset of labor in hours.	The responses were Categorizes in to four as: ‘No leakage before onset of labor’”, ‘<1 hour’, ‘1–12 hours’ and ‘>12 hours’.
Duration of labor	The time between the onset of labor to the expulsion of the foetus	Categorized in to three as: ‘<6 hours’, ‘6–12 hours’ and ‘>12 hours’.
Complications during labor	The occurrence of one or more of the following complications: excessive bleeding, mother had convulsions, breech presentation, emergency C/S and multiple delivery.	The responses were categorized as ‘Yes’ if at least any one complication and otherwise categorized as ‘No’.
Type of birth	Multiplicity of the birth (whether the delivery was multiple or singleton)	Twin births were labeled as ‘1’ and singletons were labeled as ‘0’
Sex of neonate	The sex of the neonate, both for died and alive.	Males were coded as ‘1’ and females were coded as ‘0’.
Size of neonate at birth	Size of their neonate at birth as judged by the mother as compared to other average neonates they know before.	The responses were categorized in to 5 as: ‘very small’, ‘small’ ‘average’, ‘big’ and ‘very big’ which later recoded in to three as “Small’, average’ and big’ by merging the lower two as well as the upper two categories.
Neonatal care	The minimum neonatal care packages adapted from WHO having 12 items were used to produce composite index by using PCA.	Mean score was computed for the index and those scored above or equal to the mean were categorized as having ‘good neonatal care’ and those scoring less than the mean were categorized as ‘poor neonatal care’.

### Instruments

The data were collected by using pre-tested interviewer administered structured questionnaires which were adapted from different literatures. The indicators for the wealth index were adapted from Ethiopian Demographic and Health Survey (EDHS) [5]. Indicators to measure birth preparedness and complication readiness (BP & CR) were adapted from the safe motherhood questionnaires developed by maternal and neonatal health program of Johns Hopkins Program for International Education in Gynecology and Obstetrics (JHPIEGO) [9]. Indicators for neonatal care practices were adapted from the World Health Organization (WHO) minimum neonatal care packages [10]. Data on causes of neonatal death were collected by using structured verbal autopsy questionnaire adapted from the standard VA questionnaire developed and validated by WHO, Johns Hopkins University (JHU) and London School of Hygiene and Tropical Medicine [11]. All the questionnaires were prepared in English, then translated to local languages ‘Afan Oromoo’ and Amharic and used to collect the data after back translating to English by different experts to check its consistency.

### Data collection process

As this was prospective follow up study, data were collected in three phases. First, home-to-home visit was made to enumerate pregnant women from the selected 73 clusters. Then, all the identified pregnant women were enrolled in the study as a cohort. At a baseline, data on basic socio-demography, economy and birth preparedness and complication readiness were collected. Then, just at the end of neonatal period, maternal service use (antenatal care (ANC), delivery place and attendant and postnatal care), conditions of labor, neonatal characteristics and neonatal care practices were collected. For died neonates, VAs were conducted within 15–30 days of death.

Females, who had completed 10^th^ grade or above were recruited, trained and collected the data. The VAs were conducted by two experienced females. The data collection process was supervised strictly by trained supervisors and principal investigators. To control the quality of data, in addition to training, pretest, supervision and use of local languages, the inter-item consistency of the indicators to measure the composite score of wealth index, BP & CR and neonatal care practices were checked by using Chronbach-alpha at 0.7 cut-off points.

### Data management and analysis

The collected data were coded and entered into Epidata V.3.1 to minimize logical errors and design skipping patterns. Then, the data were exported to SPSS for windows version 20.0 for cleaning, editing and analysis. Descriptive analysis was done by computing proportions and summary statistics. Socioeconomic quintiles were determined by using Principal Component Analysis (PCA). Birth preparedness and complication readiness was computed by composite indicator of five items. Similarly, neonatal care practice was determined by composite variable of 12 items by using PCA. As Jimma and Agaro town administrations were both purposefully included, the status of neonatal mortality was estimated by calculating weighted percentage based on the complex sample survey procedure to avoid underestimation.

Bivariate analysis was done by using cross-tabulation to see associations between the dependent and independent variables. Then, all variables having P-value <0.25 were considered as candidates for the final model. As multistage-clustered sampling technique was used because of the different levels of factors, mixed-effects multilevel logistic regression model was used by using STATA 13. This model was preferred in order to avoid the clustering effects as well as ecological fallacy. ‘Kebeles’ were considered as clusters and ‘kebele’ level variables were taken as higher-level (level 2). Neonates were nested within their family and households. As a result, neonatal individual variables, delivery conditions and household characteristics were taken as lower level (level 1) variables (Table 1).

Goodness of fit of the multilevel model was tested by the log likelihood ratio (LR) test. To evaluate the extent of the cluster variation in influencing neonatal mortality, intercept-only model was fitted as Logit (*p_ij_*)  = γ_00_+u_0j_ and Interclass Correlation Coefficient (ICC = ρ) was determined by dividing variance between groups (δ^2^µ_0_) by variance between groups (δ^2^µ_0_) plus variance within groups (δ^2^e). However, within group variance (δ^2^e) can't be directly obtained for dichotomous outcome variables; instead, it was estimated by dividing δ^2^µ_0_ by δ^2^µ_0_ plus ∏^2^/3. Then, to identify the determinant factors, the full model was fitted as: Logit(p_ij_)  = γ_00_ + γ_01_ Z_j_ + γ_10_ X_ij_ + u_0j_ + u_1j_ X_ij_. Where: Logit(p_ij_)  =  dependent variable at unit i in cluster j, X_ij_ =  individual explanatory variable in cluster j, Z_j_ =  group level explanatory variable, γ_00_ =  fixed intercept, γ_01 and_ γ_10_ =  fixed slopes and u_0j_ and u_1j_ =  random effects at level 2.

Multicollinearity between the independent variables was assessed by using variance inflation factors (VIF>10 considered as existence of multicollinearity) before interpreting the final output. However, only skill of delivery attendant (VIF = 10.9) had multicollinearity with place of delivery (VIF = 9.1, reduced to 1.8 when delivery attendant was dropped). As a result, they were included in the model alternatively by dropping the other. For the rest of the variables, the VIF was <3. In addition, cross-level two-way interactions were checked, particularly between place of residence, access to health facilities and maternal health care use (ANC, place of delivery and delivery attendants). Individual-level two-way interactions between prematurity (gestation age at birth), twin births and size of neonate at birth were checked. However, no significant interaction was detected (P>0.05 for each). The VAs were interpreted by two independent pediatricians and third pediatrician interpreted in case of disagreements.

### Ethical consideration

Ethical approval was obtained from the Institutional Review Board (IRB) of College of Health Sciences of Addis Ababa University as well as IRB of Oromia Regional State Health Bureau. Following this, formal letters and permissions were secured from all respective local administrators. Written informed consent was obtained from each respondent before actual data collection. Issues of confidentiality were maintained by removing any identifiers from the questionnaire. To protect vulnerable group, data collectors were trained to maintain confidentiality and provide necessary health information based on the need of the participants and arrange referral to health facilities for sick neonates.

## Results

### Response rate

It was planned to include a sample of 3604 neonates. However, after excluding incomplete questionnaires, abortion cases, missed-to-follow up, maternal deaths and stillbirths, a total of 3463 live-births were included in the analysis for this study making a response rate to be 96.1%.

**Figure 1:**
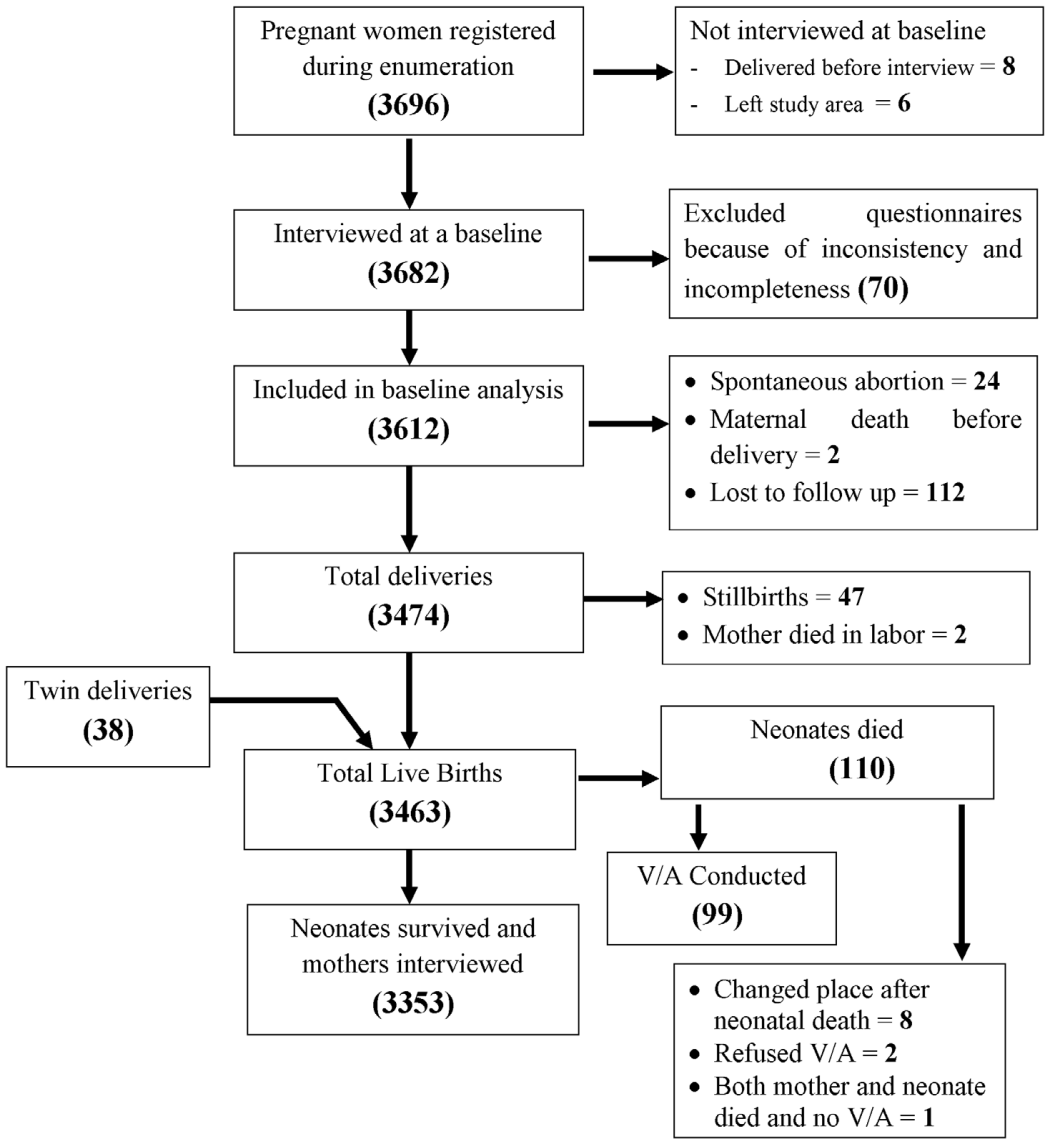
Flow-diagram of the overall study process, Jimma Zone, Southwest Ethiopia, Sept 2012-Dec 2013. This figure shows that a total of 3696 pregnant women were obtained during enumeration. After excluding 14 women because of different reasons, 3682 women were interviewed at a baseline. Again after excluding 70 incomplete and inconsistent questionnaires, 3612 were included in the analysis and enrolled in the follow up. After the follow up, total of 3472 deliveries happened; among which 38 were twins and 47 were stillbirths. From the total of 3463 live births, 110 died before 28 days.

The detail process and flow of the study is indicated below (Figure 1).

### Socio-demographic characteristics

Of the total 3463 live-births included in the analysis, 2602(75.1%) were from urban residence. Majority, 2209(63.8%), of the mothers of the neonates were in the age group of 20–29 years with a mean and standard deviation of 26.6±5.0. Oromoo was the dominant ethnic group, 3033(87.6%) and Muslim was the leading religion, 3019(87.2%). More than half of the mothers, 1871(54.0%), didn't attend any formal education. The great majority, 3280(94.7%), of the mothers were housewives and farmer was the leading occupation of their husbands, 2459(71.0%). Nearly half, 1779(51.4%), of the neonates were males with male-to-female ratio of 1.06∶1.00. The rate of twin births was 76(2.2%) (Table 2).

**Table 2:** Socio-Demographic characteristics of Respondents, Jimma Zone, Southwest Ethiopia, September 2012-December 2013 (n = 3463).

Variables	No.	%
Place of residence		
Urban	861	24.9
Rural	2602	75.1
Age (Years)		
<20	174	5.0
20–29	2209	63.8
≥30	1080	31.2
Ethnicity		
Oromo	3033	87.6
Amhara	169	4.9
Dawuro	96	2.8
Others*	165	4.7
Religion		
Musilim	3019	87.2
Orthodox	345	10.0
Protestant	99	2.9
Educational status		
No formal education	1871	54.0
Primary (1–8)	1270	36.7
Secondary (9–12)	256	7.4
>12	66	1.9
Occupation		
Housewife	3280	94.7
Employed (Gov't, NGO & Private)	78	2.2
Others‡	105	3.1
Husband's Occupation		
Farmer	2459	71.0
Employed (Gov't, NGO & Private)	376	10.8
Merchant	413	11.9
Daily laborer	190	5.5
Others‡	25	0.8
Sex of neonates		
Male	1779	51.4
Female	1684	48.6
Types of birth		
Singleton	3387	97.8
Twins	76	2.2

*Yem, Kaficho, Guraghe & Tigrie,

† Single, divorced & widowed,

‡ Merchant, student, daily laborer,

### Status of neonatal mortality

From a total of 3463 live-births, 110 died before 28 days of birth making weighted neonatal mortality rate of 35.5 (95%CI: 28.3, 42.6) per 1000 live-births (urban  = 20.0 (95%CI: 9.0, 31.0) and rural 36.2 (95%CI: 28.7, 43.7). From these, 76(69.1%) died within the first week of life making weighted early neonatal mortality rate to be 23.7 (95%CI: 18.5, 30.3). In this study, 47 stillbirths happened making a stillbirth rate and perinatal mortality rate to be 16.5 (95%CI: 12.2, 22.4) and 39.8 (95%CI: 32.9, 48.1) per 1000 births, respectively (Table 3).

**Table 3:** The status of neonatal mortality in Jimma Zone, Southwest Ethiopia, September 2012-December 2013 (n = 3463).

Events	No.	Rate/10^3^ (95%CI) (unweighted)	Rate/10^3^ (95%CI) (weighted)
Total births	3510		
Total live-births	3463		
Stillbirths	47	13.4(10.1, 17.8)	16.5(12.2, 22.4)
Early neonatal mortality	76	22.0(17.6, 27.4)	23.7(18.5, 30.3)
Late neonatal mortality	34	9.8(7.0, 13.7)	11.8(8.2, 16.9)
Perinatal mortality	123	35.1(29.5, 41.7)	39.8(32.9, 48.1)
Neonatal mortality	110	31.8(26.4, 38.2)	35.5(28.3, 42.6)

### Determinants of neonatal mortality

To evaluate the applicability of the mixed-effects multilevel logistic regression model, the ICC (ρ) was calculated in the empty model and it was found to be 0.100 indicating that 10.0% of the variation is contributed by between-cluster variation. The test of the preference of log likelihood Vs logistic regression was also strongly significant (P = 0.0003). Then, the full model was run by including all the cluster level and individual level variables and the ICC (ρ) was increased to 0.174 suggesting model improvement. This again indicated that 17.4% of the variation is attributed to cluster level variables suggesting the preference of multilevel analysis. The preference of log likelihood Vs logistic regression was again improved and strongly significant (P = 0.0001) (Table S1).

After adjusting in the final two-level mixed-effects logistic regression model, cluster level variables (place of residence, access to BEmOC and CEmOC) had non- significant associations with neonatal mortality. Similarly, among the lower level variables maternal socio demography and economy such as age, education, occupation and wealth quintiles had non-significant associations. Whereas, maternal obstetric and health care during pregnancy and delivery, delivery conditions, neonatal conditions and neonatal care practice had significant associations.

First birth order (OR = 5.45; 95%CI: 1.81, 16.40) and birth order of 5^th^ or above neonates (OR = 2.61; 95%CI: 1.43, 4.74) were more likely to die during neonatal period as compared to 2^nd^–4^th^ order. Those whose mothers had 1–3 ANC visits (OR = 0.51; 95%CI: 0.28, 0.93) and 4 or more visits (OR = 0.35; 95%CI: 0.18, 0.68) were less likely to die during neonatal period as compared to those who had no ANC visit at all. Neonates born at health centers were 43% (OR = 0.43; 95%CI: 0.17, 0.99) less likely to die during neonatal period as compared to those who were born at home. However, hospital delivery (OR = 0.73; 95%CI: 0.31, 1.70) and skilled attendants (OR = 0.57; 95%CI: 0.28, 1.16) had non-significant association as compared to home delivery and non skilled attendants, respectively. Prematurity (GA at birth <37weeks) was found to increase the likelihood of neonatal death as compared to term births (OR = 2.09; 95%CI: 1.03, 4.22). Premature and prolonged rupture of membrane before the onset of labor had increased the likelihood of neonatal death. Rupture of membrane 1–12 hours (OR = 2.71; 95%CI: 1.13, 6.53) and >12 hours (OR = 7.74; 95%CI: 2.27, 26.38) before the onset of labor had significantly higher risk of neonatal death as compared to rupture of membrane after the onset of labor.

The occurrence of obstetric complications during labor (OR = 6.77; 95%CI: 3.82, 12.00) and twin births (OR = 8.21; 95%CI: 3.46, 19.47) were among the strong predictors of neonatal mortality. Similarly, small size (OR = 1.95; 95%CI: 1.11, 3.42) and big size (OR = 10.73; 95%CI: 5.65, 20.37) at birth were found to increase the likelihood of neonatal death as compared to average size neonates. Not having good comprehensive neonatal care practice was the other strong predictor of neonatal mortality (OR = 10.36; 95%CI: 5.13, 20.94) (Table 4).

**Table 4:** Multilevel analysis of factors associated with neonatal mortality, Jimma Zone, Southwest Ethiopia, Sept 2012-Dec 2013.

Variables	Neonatal Mortality			Crude OR(95%CI)	Adjusted OR(95%CI)
	Died (n = 110) n(%)	Survived (n = 3353) n(%)	Total (n = 3463) n(%)		
**Higher level variables**					
Place of residence					
Urban	18(2.1)	843(97.7)	861(100.0)	1.00	1.00
Rural	92(3.5)	2510(96.5)	2602(100.0)	1.72(1.03, 2.86)	1.08(0.33, 3.57
Average distance from Health centre (on foot)					
≤2 hours	77(3.1)	2417(96.9)	2494(100.0)	1.00	1.00
>2 hours	33(3.4)	936(96.6)	969(100.0)	1.12(0.73,1.68)	1.06(0.49, 2.32)
Average distance from Hospital (on foot)					
≤2 hours	10(1.9)	519(98.1)	529(100.0)	1.00	1.00
>2 hours	100(3.4)	2834(96.6)	2934(100.0)	1.83(0.95, 3.53)	1.92(0.34, 4.20)
**Level-1 variables**					
Age of mother at birth					
15–19	3(1.7)	171(98.3)	174(100.0)	1.00	1.00
20–29	64(2.9%)	2145(97.1)	2209(100.0)	1.70(0.53, 5.47)	3.39(0.81, 14.21)
>29	43(4.0)	1037(96.0)	1080(100.0)	2.36(0.73, 7.70)	4.04(0.87, 18.71)
Educational status of mother					
Illiterate	69(3.7)	1802(96.3)	1871(100.0)	1.00	1.00
Primary(1–8)	37(2.9)	1233(97.1)	1270(100.0)	0.78(0.52, 1.18)	0.96(0.56, 1.66)
Secondary or above (≥9)	4(1.2)	318(98.8)	322(100.0)	0.33(0.12, 0.91)	0.52(0.13, 2.13)
Mother's occupation					
Unemployed (housewife)	107(3.3%)	3173(96.7)	3280(100.0)	1.00	1.00
Employed	3(1.6)	180(98.4)	183(100.0)	0.50(0.16, 1.58)	0.41(0.10, 1.81)
Father's occupation					
Farmer	86(3.5)	2372(96.5)	2458(100.0)	1.00	1.00
Employed	6(1.6)	370(98.4)	376(100.0)	0.45(0.19, 1.03)	1.32(0.36, 4.84)
Merchant	18(2.9)	611(97.1)	629(100.0)	0.81(0.49, 1.36)	1.92(0.79, 4.66)
Wealth quintiles					
First quintile	30(4.4)	648(95.6)	678(100.0)	1.00	1.00
Second quintile	24(3.4)	679(96.6)	703(100.0)	0.76(0.44, 1.32)	0.87(0.43, 1.74)
Third quintile	24(3.5)	665(96.5)	689(100.0)	0.78(0.45, 1.35)	1.02(0.51, 2.04)
Fourth quintile	17(2.4)	680(97.6)	697(100.0)	0.54(0.30, 0.99)	1.01(0.47, 2.15)
Fifth quintile	15(2.2)	681(97.8)	696(100.0)	0.48(0.25, 0.89)	0.72(0.32, 1.62)
Birth order					
1st	28(3.8)	703(96.2)	731(100.0)	2.00(1.20, 3.28)	5.45(1.81, 16.40)
2nd–4th	36(2.0)	1793(98.0)	1829(100.0)	1.00	1.00
5th or above	46(5.1)	857(94.9)	903(100.0)	2.67(1.72, 4.17)	2.61(1.43, 4.74)
Preceding birth interval					
<24 months	8(3.1)	247(96.9)	255(100.0)	1.00	1.00
24–48 months	62(2.9)	2095(97.1)	2157(100.0)	0.91(0.43, 1.93)	1.34(0.49, 3.65)
>48 months	12(3.8)	308(96.2)	320(100.0)	1.20(0.48, 2.99)	2.23(0.66, 7.57)
Nuliparous	28(3.8)	703(96.2)	731(100.0)	NA	NA
Birth preparedness plan					
Prepared	28(2.3)	1174(97.7)	1202(100.0)	1.00	1.00
Not prepared	82(3.6)	3353(96.8)	3463(100.0)	1.58(1.02, 2.44)	1.00(0.55, 1.84)
Frequency of ANC					
No visit at all (0)	32(3.9)	787(96.1)	819(100.0)	1.00	1.00
1–3 visits	44(3.1)	1362(96.9)	1406(100.0)	0.80(0.50, 1.26)	0.51(0.28, 0.93)
≥4 visits	34(2.7)	1204(97.3)	1239(100.0)	0.70(0.43, 1.14)	0.35(0.18, 0.68)
Place of delivery					
Home	83(3.5)	2316(96.5)	2399(100.0)	1.00	1.00
Hospital	17(3.9)	418(96.1)	435(100.0)	1.14(0.67, 1.93)	0.73(0.31, 1.70)
Health centre	10(1.6)	619(98.4)	629(100.0)	0.45(0.23, 0.87)	0.43(0.17, 0.99)
Attendant of delivery					
Non skilled	83(3.5)	2316(96.5)	2399(100.0)	1.00	1.00
Skilled	27(2.5)	1037(97.5)	1064(100.0)	0.73(0.47, 1.13)	0.57(0.28, 1.16)
Gestation age at birth					
Term (≥37 weeks)	90(2.9)	3030(97.1)	3120(100.0)	1.00	1.00
Preterm (<37 weeks)	20(5.8)	323(94.2)	343(100.0)	1.09(1.27,3.43)	2.09(1.03, 4.22)
Premature rupture of membrane					
No(during labor)	84(2.9)	2773(97.1)	2857(100.0)	1.00	1.00
<1 hour before labor	7(1.5)	470(98.5)	477(100.0)	0.49(0.23, 1.07)	0.40(0.16, 1.01)
1–12 hours before labor	12(12.4)	85(87.6)	97(100.0)	4.66(2.45, 8.86)	2.71(1.13, 6.53)
>12 hours before labor	7(21.9)	25(78.1)	32(100.0)	9.24(3.89, 21.97)	7.74(2.27, 26.38)
Duration of labor					
<6 hours	36(1.8)	1931(98.2)	1967(100.0)	1.00	1.00
6–12 hours	45(4.8)	885((95.2)	930(100.0)	2.73(1.75, 4.26)	1.47(0.83, 2.58)
>12 hours	29(5.1)	537(94.9)	566(100.0)	2.90(1.76, 4.78)	0.84(0.41, 1.72)
Complication during labor					
No	40(1.5)	2645(98.5)	2685(100.0)	1.00	1.00
Yes	70(9.0)	708(91.0)	778(100.0)	6.54(4.40, 9.73)	6.77(3.82, 12.00)
Types of birth					
Singleton	94(2.8)	3293(97.2)	3387(100.0)	1.00	1.00
Twins	16(21.1)	60(78.9)	76(100.0)	9.34(5.19, 16.83)	8.21(3.46, 19.47)
Sex of neonate					
Male	69(3.9)	1710(96.1)	1779(100.0)	1.00	1.00
Female	41(2.4)	1643(97.6)	1684(100.0)	0.62(0.42, 0.92)	0.77(0.48, 1.24)
Size of neonate at birth					
Small	41(5.3)	736(94.7)	777(100.0)	3.37(2.16, 5.25)	1.95(1.11, 3.42)
Average	40(1.6)	2419(98.4)	2459(100.0)	1.00	1.00
Big	29(12.8)	198(87.2)	227(100.0)	8.86(5.38, 14.60)	10.73(5.65, 20.37)
Neonatal care practice					
Good	19(1.0)	1806(99.0)	1825(100.0)	1.00	1.00
Poor	91(5.6)	1547(94.4)	1638(100.0)	5.59(3.39, 9.17)	10.36(5.13, 20.94)

NA =  Not Applicable.

### Causes of neonatal mortality

**Figure 2:**
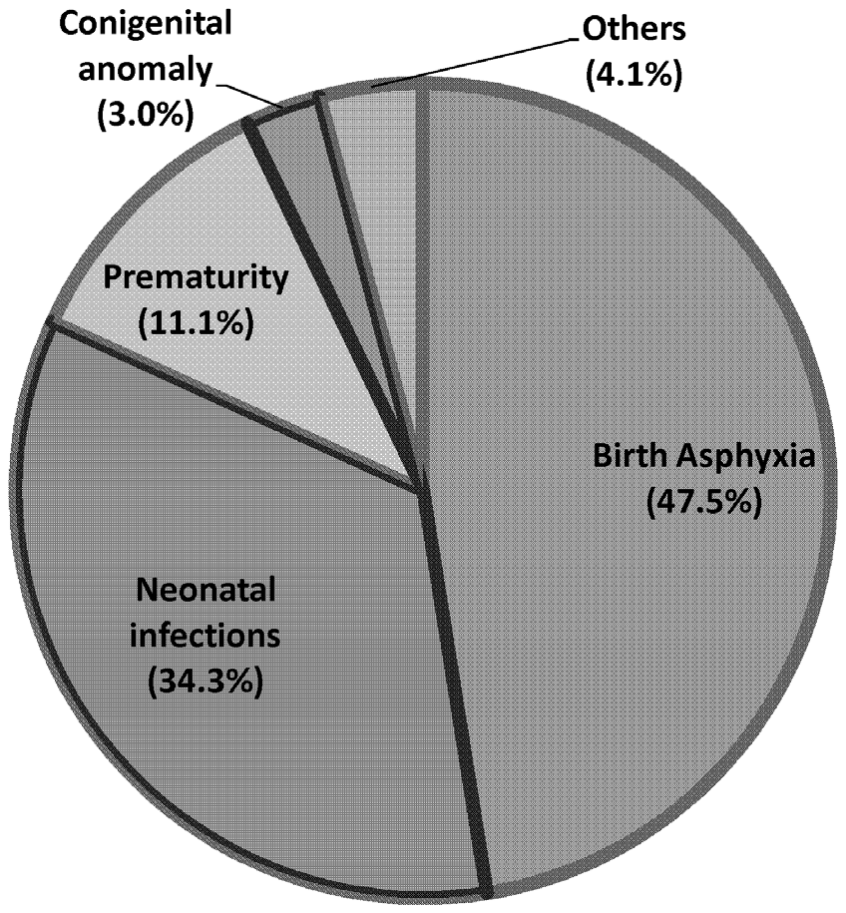
Causes of neonatal death, Jimma Zone, Southwest Ethiopia, Sept 2012–Dec 2013. This figure shows that birth asphyxia (47.5%), neonatal infections (34.3%) and prematurity (11.1%) were the three leading cause of neonatal death accounting for nearly 93%.

Out of the total 110 neonatal deaths occurred, VAs were conducted for 99 cases. Initially, the two interpreting physicians agreed on the most probable cause of death for 86 out of 99 cases, which was 86%. For 6 (6.1%) of the cases, first and second most probable causes were exchanged between the physicians making the overall agreement rate to be 93%. As a result, third physician interpreted the 13 cases and agreed with either of the physicians in 11 cases and the rest 2 cases were not agreed upon and classified as unspecified cause of death. With this, birth asphyxia (47.5%), neonatal infections (34.3%) and prematurity (11.1%) were the three leading causes of neonatal mortality (Figure 2).

## Discussions

The status of neonatal mortality rate in this study is 35.5 (95%CI: 28.3, 42.6) per 1000 live births. This is similar with the finding of the EDHS 2011 in which NMR was 37.0 (95%CI: 33.7, 40.3) [5]. However this is higher than the findings of other countries with high neonatal mortality like India (19.5) [12] and Indonesia (23.8) [13]. This clearly indicated that the situation of neonatal mortality is still high and non-progressing suggesting targeted interventions by all partners at different levels.

In this study, 63 (57.3%) and 76 (69.1%) out of the 110 died neonates occurred on the date of birth and in the 1^st^ week of life, respectively making early NMR to be 23.7 per 1000 live-births. This is in line with other prior studies in which more than three-quarter of neonatal deaths occurred in the first week of life [13]–[17]. The reason could be majority of neonatal mortality in developing countries are related to conditions of labor, intra-partum and the immediate newborn care practices. Moreover, the major causes of death are birth asphyxia, early neonatal infections and prematurity. This again clearly pointed out that neonatal survival interventions have to target the intra-partum as well as immediate and early neonatal periods.

In this study, higher-level factors (place of residence, access to health centre and hospital) were found to be less important in predicting neonatal mortality. Similarly, lower level factors related to basic socio-demography and economy were less important. Instead, lower level factors related to maternal and neonatal complications and care before, during and after delivery were the most important predictors of neonatal mortality. This finding is consistent with some other prior studies in which higher level variables had less importance in determining neonatal mortality [14], [16], [18], [19]. However, this finding is in contrast to the findings of study done in rural India in which both the higher and lower level factors were equally important [12]. This pointed out that the higher-level factors that are less amenable to short term interventions, have to be considered as distant factors and special focus needs to be given to the immediate proximal factors of neonatal mortality which are more feasible to be intervened.

First birth order and birth order of five or above were found to increase the likelihood of neonatal mortality by more than five and two times respectively. This may be due to high risk of occurrence of complications during delivery among nuliparous and grandmultiparous mothers. In this study, having ANC visit and giving birth at health center were found to decrease neonatal mortality significantly. This is consistent with previous study conducted in Ethiopia and other countries [13], [20]. This may be because; during ANC visits, necessary health condition of mothers can be screened and treated earlier. Moreover, health facility delivery is very necessary in detecting complications earlier and providing clean and safe delivery.

In the contrary, delivery at hospital and skilled attendants at birth had no significant association with neonatal mortality. Similarly, in the systematic review and meta-analysis we conducted before this study, in 9 of the 19 studies included, health facility delivery had no significant effect on neonatal mortality [21]. Similar findings have been reported in some other studies in the country and abroad [15], [22]. This may be because, majority of hospital deliveries in developing countries, including Ethiopia, are as a result of self referral after all attempts failed. In this study for example, 43.5% of hospital deliveries were because of occurrence of problems during labor. This highlighted the importance of addressing the first and the second delays. This means, giving birth at hospital attended by skilled attendant may not be a guarantee to avert neonatal mortality unless the woman comes to the right health facility at the right time. The other possible explanation could be the low coverage of hospital delivery and skilled attendant at birth that lead to low power to detect significant effects.

Intra-partum conditions, neonatal conditions and immediate neonatal care practices were the most important determinants of neonatal mortality in this study. Prematurity (<37weeks of GA at birth) and the occurrence of maternal complications during labour were found to increase the likelihood of neonatal mortality by two times and nearly seven times, respectively. Similarly, rupture of membrane within 1–12 hours and >12 hours before the onset of labor had increased neonatal mortality by nearly three times and eight times, respectively. Twin births was also increased the risk of neonatal mortality by eight times as compared to singletons. Small size and big size at birth were increased the likelihood of neonatal mortality by about two and eleven times, respectively. Neonates having poor neonatal care were about ten times more likely to die during neonatal period as compared to those who received good comprehensive neonatal care.

These findings are consistent with other previous studies conducted in the country and abroad in which the intra-partum and neonatal conditions were found to be the important predictors of neonatal mortality [14], [15], [17], [18], [19], [22]. This may be explained by the fact that premature and twin births are more likely to be under-weight and more prone to complications and infections. Similarly, long staying PROM and intra-partum complications increase the risk of infections and birth asphyxia. Thus, provision of comprehensive neonatal care including clean cord care, thermal care and appropriate feeding have the potential to avert some of these risks significantly.

In line with this, the verbal autopsy identified birth asphyxia, neonatal infections and prematurity as the major causes of neonatal mortality accounting for 93%. This is also in line with other prior studies where the three causes accounted for more than four-fifth of neonatal mortality [14], [23]–[25]. While infection is the leading cause of neonatal mortality in many studies in developing countries [23], birth asphyxia was the leading cause of death (47.5%) in this study. This finding may be explained by the high proportion of home deliveries in the absence of skilled attendants. Knowledge and skill deficiencies in prevention, diagnosis and management by unskilled attendants might have contributed to perinatal complications associated with neonatal asphyxia in these communities. The association between elevated neonatal mortality and home deliveries by unskilled attendants highlights the importance of delivery care education to local service providers.

As a programmatic implication, this study pointed out that targeting individual level factors like intra-partum conditions and the immediate neonatal period can significantly improve neonatal survival. Moreover, targeting the three major causes of death (birth asphyxia, neonatal infections and prematurity) can avert more than nine in ten of neonatal deaths.

This study may have its own limitation in that some of the medical terms were difficult to translate exactly to local languages, which might have affected the respondents' understanding. To reduce this limitation, local language experts translated the instruments. In addition, local data collectors who were fluent in local languages and familiar with local terms collected the data. The verbal autopsy was based on mothers' report of signs and symptoms concerning the underlying causes of neonatal death. This may not be as specific as the clinical diagnosis in identifying the exact cause of death. Therefore, future researches need to consider additional clinical and laboratory-based identification of causes of neonatal death by including facility based data for better interventions. In addition, the verbal autopsies were conducted within 15–30 days of neonatal death which might lead to recall bias of the exact conditions of death of the neonates. To address this, standardized and validated questionnaire was adapted in order that the mothers remember the conditions and report more valid data.

## Conclusions

This study revealed that the status of neonatal mortality in the study area is still high. The great majority of neonatal deaths occurred in the first week of life. Higher-level variables were found to be less important in determining neonatal mortality. Lower (individual) level variables were found to be important determinants of neonatal mortality. Birth order, ANC visits, place of delivery, prematurity, PROM, complication during labor, twin births, size of neonate at birth and neonatal care practices were identified as determinants of neonatal mortality. Birth asphyxia, neonatal infections and prematurity were identified as the leading causes of neonatal mortality accounting for more than 90%. Ensuring the continuum of care from pregnancy through delivery to the immediate postnatal period should be in place so as to address neonatal mortality in the study area. Specifically, increasing adequate ANC visits and health facility delivery by addressing the first and second delays are very crucial. Provision of comprehensive neonatal care such as cord care, thermal care and early initiation of breast-feeding are recommended.

## Supporting Information

Figure S1 **Schematic presentation of sampling method.** This figure shows the multistage clustered sampling methods based the proportional allocation to the size of the population.
(TIF)

Table S1 **Parameter coefficients and test of goodness-of-fit of the mixed effect multilevel model, in Jimma Zone, Southwest Ethiopia, September 2012-December 2013.** This table shows the parameter estimates of the multilevel logistic regression, including the fixed effects, random effect at level 2, the Infraclass correlation Coefficient, LR test and level of significance both in the empty-model and full model.
(DOCX)
