# Supplementary material for: Determinants and Causes of Neonatal Mortality in Jimma Zone, Southwest Ethiopia: A Multilevel Analysis of Prospective Follow Up Study
Source: PLoS One. 2014 Sep 18;9(9):e107184. doi: 10.1371/journal.pone.0107184 (PMC4169420; doi:10.1371/journal.pone.0107184)
Supplement: Table S1 — Parameter coefficients and test of goodness-of-fit of the mixed effect multilevel model, in Jimma Zone, Southwest Ethiopia, September 2012-December 2013. This table shows the parameter estimates of the multilevel logistic regression, including the fixed effects, random effect at level 2, the Infraclass correlation Coefficient, LR test and level of significance both in the empty-model and full model. (DOCX) [file pone.0107184.s002.docx]

**Table S1.** Parameter coefficients and test of goodness-of-fit of the mixed effect multilevel model, in Jimma Zone, Southwest Ethiopia, September 2012-December 2013

| Models | Fixed intercept  -cons(95%CI) | Random effect as Level-2 variance var(-cons (95%CI)) | Intra-class Correlation Coefficient: ICC(ρ) | Log likelihood (LR)  (deviance) | Significance of  LR test Vs Logistic regression (P-value) |
| --- | --- | --- | --- | --- | --- |
| Empty model | -3.63(-3.92, -3.34) | 0.34(0.14, 0.86) | 0.100 = 10% | -481.92 | 0.0003 |
| Full model | -7.27(-9.71, -4.83) | 0.69(0.27, 1.76) | 0.174 = 17.4% | -325.61 | 0.0001 |
